# Failure to Eradicate *Isospora belli* Diarrhoea Despite Immune Reconstitution in Adults with HIV - A Case Series

**DOI:** 10.1371/journal.pone.0042844

**Published:** 2012-08-06

**Authors:** Tom H Boyles, John Black, Graeme Meintjes, Marc Mendelson

**Affiliations:** 1 Division of Infectious Diseases and HIV Medicine, Department of Medicine, Groote Schuur Hospital, University of Cape Town, Cape Town, South Africa; 2 GF Jooste Hospital, Manenberg, Cape Town, South Africa; 3 Institute of Infectious Diseases and Molecular Medicine, Cape Town, South Africa; University of Pittsburgh, United States of America

## Abstract

*Isospora belli* causes diarrhoea in patients with AIDS. Most respond to targeted therapy and recommendations are that secondary prophylaxis can be stopped following immune reconstitution with ART. We report eight cases of chronic isosporiasis that persisted despite standard antimicrobial therapy, secondary prophylaxis, and good immunological and virological response to ART. Median CD4 nadir was 175.5 cells/mm^3^ and median highest CD4 while symptomatic was 373 cells/mm^3^. Overall 34% of stool samples and 63% of duodenal biopsy specimens were positive for oocytes. Four patients died, two remain symptomatic and two recovered. Possible explanations for persistence of symptoms include host factors such as antigen specific immune deficiency or generalised reduction in gut immunity. Parasite factors may include accumulating resistance to co-trimoxazole. Research is required to determine the optimum dose and duration of co-trimoxazole therapy and whether dual therapy may be necessary. Mortality was high and pending more data we recommend extended treatment with high-dose co-trimoxazole in similar cases.

## Introduction

The coccidian parasite *Isospora belli* is among the most frequently identified causes of chronic diarrhoea in patients with AIDS [1]. It predominates amongst patients with CD4 count <200 cells/mm^3^ [2] and the incidence has decreased since the introduction of combination antiretroviral therapy (ART) [3]. Treatment with co-trimoxazole is effective in ART naive HIV-infected patients [4] although relapses are common without secondary prophylaxis [5]. In cases of co-trimoxazole intolerance, second line drug options include ciprofloxacin [6], pyrimethamine [7] and nitazoxanide [8]. It is recommended that secondary prophylaxis can be stopped when CD4 count is >200 cells/mm^3^ for greater than 6 months on ART although this has not been validated in clinical trials [9]. There are no reports of adult HIV-infected patients who fail to eradicate *Isospora belli* following immune reconstitution and chronic isosporiasis has only rarely been reported in immunocompetent hosts [7]. The standard of care for *Isospora belli* in South Africa is co-trimoxazole 960 mg 6 hourly for 10 days followed by co-timoxazole 960 mg daily until CD4 count >200 cells/mm^3^. We report 8 cases of recurrent, chronic isosporiasis in HIV-infected adults on ART in whom HIV replication was suppressed to undetectable levels and in whom immunological evidence of immune reconstitution was demonstrated.

## Methods

Local databases of patients referred to the infectious diseases services at GF Jooste and Groote Schuur hospitals in Cape Town were searched to identify patients with HIV infection and chronic diarrhoea. Paper and electronic clinical records were then examined to identify those with >1 stool or biopsy sample positive for *Isospora belli* oocytes, persistent symptoms despite appropriate targeted treatment and virological suppression and immune reconstitution on ART.

Local research ethics committee approval was granted to analyse data held on a departmental database. Information reflecting the identity of the patients was removed.

## Results

From January 2005 to January 2010 eight patients meeting the search criteria were identified. Five were female and median age at presentation was 34 years; their clinical course is summarised in table 1 and a detailed history of 2 cases is described below. Chronic isosporiasis was confirmed by the presence of oocytes on multiple occasions in stool examined by modified acid-fast staining or duodenal biopsy specimen examined by standard H&E staining. In total 34% (23/67) of stool samples and 63% of (10/16) duodenal biopsies were positive for oocytes. Alternative causes of diarrhoea were excluded by multiple negative stool cultures for *Salmonella* and *Shigella* species and negative *Clostridium difficile* toxin testing. In addition 7 patients underwent duodenal biopsy, often on multiple occasions, with no alternative cause for chronic diarrhoea being identified. All patients achieved an undetectable HIV viral load (<50 copies/ml) while remaining symptomatic. Four patients never had an HIV viral load >50 copies/ml despite regular measurement. Three patients had only low level intermittent viraemia (maximum 2780 copies/ml) and one experienced high level virological failure (306671 copies/ml) but remained symptomatic despite subsequently suppressing HIV viral load to <50 copies/ml. Median CD4 nadir was 175.5 cells/mm^3^ and all patients were symptomatic despite sustained improvement in CD4 count (median increase 227 cells/mm^3^). The highest CD4 count at which a patient was symptomatic was 1013 cells/mm^3^ and the median of the highest CD4 count at which patients were symptomatic was 373 cells/mm^3^. Four patients died of complications of chronic diarrhoea and 2 remain symptomatic. Two patients eventually recovered but had been symptomatic for 2 years despite adequate targeted treatment, virological suppression and immune recovery.

**Table 1:** Clinical summary of 8 patients with chronic isosporiasis despite immunological and virological response to ART.

Case number	1	2	3	4	5	6	7	8
CD4 nadir, cells/mm^3^	32	210	281	52	221	215	141	71
Anti-retroviral regimens used	D3E/D3L/r	D3N/D3E/A3E	D3E	A3N	Tru At^r^/.Tru L^r^	D3E/.T3L^r^	D3E	D3N
Maximum CD4 count while symptomatic, cells/mm^3^ (duration of ART, months)	237(29)	1013 (60)	412 (6)	327 (24)	659 (48)	464 (17)	334 (25)	265 (23)
Any HIV viral load >50 copies/ml, (months of ART)	1000 (6)	None	86 (11) 400 (13)	None	2780 (10) 127 (48)	None	None	306671 (19)
Number of HIV viral load measurements <50 copies/ml §	3	7	2	10	8	5	2	3
Total duration of ART, years	4	5	2	7	5	3.5	2.3	2
Maximum secondary prophylaxis	CTX 1920 mg b.i.d. plus CPN 500 mg b.i.d.	CTX 1920 mg b.i.d.	CTX 960 mg b.i.d.	CTX 960 mg b.i.d.	CTX 960 mg b.i.d.	CTX 1920 mg b.i.d.	CTX 1920 mg b.i.d. plus CPN 500 mg b.i.d.	CTX 1920 mg b.i.d. plus CPN 500 mg b.i.d.
Hospital admissions for diarrhea n, (total days in hospital)	18 (151)	10 (60)	10 (81)	0	1 (8)	1 (7)	14 (71)	3 (18)
Stool samples*, n (% positive)	13 (54)	12 (8)	5 (40)	2 (100)	6 (50)	8 (50)	11 (9)	8 (50)
Duodenal biopsies, n (% positive)	3 (67)	3 (100)	3 (67)	1 (0)	2 (100)	0	3 (33)	1 (0)
Outcome	Died, complications of chronic diarrhoea	Died, complications of chronic diarrhoea	Died, complications of chronic diarrhoea	Symptoms resolved after 2 years. Currently well.	Persistent diarrhea and weight loss	Symptoms resolved after 2 years. Currently well.	Lost to follow-up presumed dead	Persistent diarrhea and weight loss. Now virologically suppressed

§ Represents measurements approximately every 6 months after ART initiation according to South African public sector guidelines applicable at the time

* All sample examined by modified acid-fast staining

CTX  =  co-trimoxazole, CPN  =  ciprofloxacin, D  =  stavudine, 3  =  lamivudine, A  =  zidovudine, E  =  efavirenz, N  =  nevirapine L^r^  =  lopinovir/ritonavir,

Tru  = tenovovir/emtracitabine, At^r^  =  atazanavir/ritonavir

### Case 1

A 33 year old HIV-infected woman with a CD4 count of 32 cells/mm^3^ weighing 52 kg, presented with 3 months of watery diarrhoea. No cause was found despite investigations including multiple stool samples examined with modified acid-fast staining. Stavudine, lamivudine and efavirenz were started 2 months later, with efavirenz being switched to lopinavir/ritonavir following a hepatitis B flare at 3 months. Despite ART, profuse watery diarrhoea continued and after 4 months she was admitted to hospital for rehydration, weighing 44 kg. *Isospora belli* oocytes were observed in stool and treatment with co-trimoxazole at 1920 mg b.i.d was successful. She gained weight to 48 kg although her symptoms returned 3 months later, when oocytes were again identified in stool, despite secondary prophylaxis with co-trimoxazole 960 mg daily. At this time, her CD4 count had risen to 174 cells/mm^3^ and HIV viral load was 1000 copies/ml. Repeat episodes of severe diarrhoea with electrolyte disturbance continued despite secondary prophylaxis; 1 year after starting ART she weighed 38 kg. She required inpatient treatment for diarrhoea and dehydration on 18 occasions over 4 years. Each time, she improved on treatment, which included the use of single drug therapy or combinations of oral or intravenous co-trimoxazole, ciprofloxacin, albendazole and nitazoxanide. She relapsed on each occasion, despite secondary prophylaxis with co-trimoxazole 960 mg b.i.d with or without ciprofloxacin 500 mg b.i.d. She was supported with intravenous fluids, electrolyte supplementation and required total parenteral nutrition on several occasions. Diarrhoea was unlikely to be a side-effect of lopinavir/ritonavir as she remained asymptomatic between acute episodes while continuing this medication. Her CD4 was consistently >150 cells/mm^3^, peaking at 237 cells/mm^3^ after 20 months of ART. HIV viral load was consistently <50 copies/ml from 12 months of ART. She died from complications of chronic diarrhoea 4 years after starting treatment for isosporiasis.

### Case 2

A 33 year old HIV-infected woman with a CD4 count of 210 cell/mm^3^ was treated with co-trimoxazole for microbiologically-proven *Isospora belli* in stool and started on secondary prophylaxis. Stavudine, lamivudine and nevirapine was started, but her gastro-intestinal symptoms never completely settled despite secondary prophylaxis with co-timoxazole at doses between 960 mg o.d. and 1920 mg b.i.d. CD4 count rose to 729 cells/mm^3^ and HIV viral replication was suppressed after 6 months of ART. After 2 years of intermittent symptoms, when her weight was 43 kg she was referred for gastroscopy and *Isospora belli* was observed in duodenal biopsy. She deteriorated and diarrhoea became more continuous; over the following 30 months she was admitted to hospital with increasing frequency. *Isospora belli* was observed in duodenal biopsies on 2 further occasions, but was absent from multiple stool samples taken at the same time. Co-trimoxazole, ciprofloxacin, paromomycin, pyrimethamine and nitazoxanide were all tried for relapses, with good initial response, but subsequent relapse on a combination of high dose co-trimoxazole and ciprofloxacin 500 mg b.i.d. secondary prophylaxis. Her weight dropped to 32 kg and she required 6 admissions in a 4 month period. Her clinical course was complicated by a deep vein thrombosis, major depressive episode, and hypocalcaemia-induced tetany requiring intubation and ICU admission. ART was continued throughout. HIV viral load recordings were consistently <50 copies/ml and her CD4 count peaked at 1013 cells/mm^3^, 5 years after starting ART. Five years after her first presentation, she died at home having declined hospital admission for a further episode of severe diarrhoea.

## Discussion

To our knowledge, this case series represents the first description of chronic, relapsing isosporiasis in HIV-infected adults despite evidence of reconstitution of CD4 T-cell count and suppression of HIV replication on ART. We think that Immune Reconstitution Inflammatory Syndrome (IRIS) is an unlikely explanation for our observations given the absence of a disproportionate inflammatory reaction when initiating ART. Our experience was of considerable morbidity necessitating multiple hospital admissions and high mortality of up to 50%. Depression was a common consequence.

There is no accepted gold standard method for diagnosing isosporiasis and the sensitivity of diagnostic tests is therefore unknown. However, we believe observing oocytes in stool or duodenal biopsy has a specificity approaching 100%. Our experience was of multiple negative stool examinations in patients even during acute episodes and when duodenal biopsy was positive; in total only 34% of stool samples were positive. This suggests that stool microscopy is insensitive and of limited use in excluding isosporiasis. Duodenal biopsies were more likely to be positive in our patients, in total 63% were positive, but duodenal biopsy is not routinely available in low resource settings where most patients with HIV live. A molecular test for identifying *Isospora belli* DNA in stool has been developed recently with promising results although prospective studies on clinical specimens are lacking [10].

Isosporiasis is typically associated with CD4 counts <200 copies/mm^3^, but our observation was that whilst a low CD4 nadir may be necessary for infection to become established, this case series represents a sub-group of patients who failed to clear the infection following reconstitution of T cell numbers. We hypothesize that several factors may contribute to this phenomenon. There may be a failure of recovery of the antigen-specific immune response to *Isospora belli* alone, as despite recurrent clinical episodes related to this specific infection, our patients did not manifest other opportunistic infections which would indicate a broader functional defect in T cell immunity. ART is known to lead to substantially delayed, and only partial restoration of gastrointestinal CD4 T cells [11] and therefore impaired gut immunity allowing persistent infection or re-infection with *Isospora belli* is a possible mechanism. Drug malabsorption is another possible factor although each patient responded adequately to ART and initial oral therapy in hospital suggesting otherwise. However, specific malabsorption of co-trimoxazole cannot be ruled out. Poor compliance with secondary prophylaxis was not judged to be a contributor, due to pill count monitoring and adherence counselling. Therapeutic drug monitoring was not performed, hence anomalies in drug pharmacokinetics cannot be ruled out.

Drug resistance to co-trimoxazole may have played a role in relapse. Acute episodes of diarrhoea generally responded to standard co-trimoxazole doses, arguing against resistance, although intermediate susceptibility of strains in patients who relapse may explain our observations. Unizoic cysts of *Isospora belli* have been described in the lymphoid tissue of a patient with AIDS [12] and lymph nodes may represent a site of chronic infection. Parasite sequestration in immune privileged sites may also play a role.

Prospective studies have shown the safety of discontinuing secondary prophylaxis against *Pneumocystis jirovecii* and *Cryptococcus neoformans* in patients with sustained improvements in CD4 counts [13]. Similar studies of patients with *Isospora belli* infection are lacking and our data suggest that similar guidelines may not be safe, at least in a sub-group of patients who remain symptomatic.

The research priority in chronic isosporiasis is to determine the optimal dose and duration of the co-trimoxazole, the most efficacious drug. The efficacy of alternative treatments including pyrimethamine and nitazoxanide must be determined and combination therapy, possibly for extended periods, should be assessed in non-responders. In similar patients we currently recommend co-trimoxazole 1920 mg b.i.d. continued for up to 2 years while monitoring for myelotoxicity.

We have managed several similar cases of chronic diarrhoea in HIV patients with immune restoration and virological suppression in rural primary care facilities, who despite negative stool samples (endoscopy and biopsy unavailable) have responded to empiric treatment for *Isospora belli*. Chronic isosporiasis was likely in these cases, so clinical diagnosis may be necessary in areas where duodenal biopsy is not routinely available.

There are several limitations to our study. Detailed analysis of strains from these patients was not undertaken, so we are unable to differentiate between reactivation of clinical disease with the same strain or re-infection with another strain. There are no accurate data for the incidence of isospora diarrhoea in our setting so we are unable to directly compare our patients with a group who were successfully treated. However, a survey of laboratory reports over 2 ½ years at GF Jooste hospital found 36 individuals with at least 1 stool sample positive for isospora oocytes. Three of these are included in our case series but the outcome of the others is unknown (unpublished data). The ability to exclude all other causes of diarrhoea was limited by resources. However multiple stool cultures in all patients and small bowel biopsies in most patients failed to reveal an alternative cause.

## Conclusions

We have described a case series of chronic isosporiasis due either to relapse or re-infection, in patients with good immunological and virological response to ART. Stool microscopy was often negative and although duodenal biopsy seemed to perform better, this investigation is rarely available outside of the major tertiary hospitals in Africa. The mortality rate in our case series was high, suggesting that new approaches to treatment such as long term intravenous therapy need to be assessed in this sub-set of patients.
